# Arginase-1 Is Responsible for IL-13-Mediated Susceptibility to *Trypanosoma cruzi* Infection

**DOI:** 10.3389/fimmu.2018.02790

**Published:** 2018-11-29

**Authors:** Mahin Abad Dar, Christoph Hölscher

**Affiliations:** Infection Immunology, Research Center Borstel, Borstel, Germany

**Keywords:** Chagas disease, interleukin-13, arginase-1, *Trypanosoma cruzi*, macrophage-cell

## Abstract

Arginase-1 (Arg-1) is a marker for alternatively activated macrophages (AAM) and is mainly induced by the type 2 cytokines interleukin (IL)-4 and IL-13 through the common IL-4 receptor-alpha (Rα) subunit. Both, Arg-1 and AAM undermine macrophage effector functions against intracellular parasites and are therefore implicated in the susceptibility to infection with *Trypanosoma cruzi*, the causative agent of Chagas' disease. However, the involvement of Arg-1 in promoting intracellular replication of *T. cruzi* in AAM has not been proven so far *in vivo*. Because Arg-1 is only moderately expressed in *T. cruzi*-infected wildtype mice, we elucidated the role of Arg-1 and AAM during infection in IL-13-overexpressing (IL-13^tg^) mice, which are characterized by an inflammation-induced development of AAM and an accompanied elevated expression of Arg-1. In comparison to wildtype littermates, IL-13^tg^ mice were highly susceptible to *T. cruzi* infection with enhanced parasitemia and impaired survival. Importantly, *T. cruzi*-infected IL-13^tg^ mice developed an elevated alternative macrophage activation with increased arginase activity. To proof the hypothesis, that Arg-1 accounts for the increased susceptibility of IL-13^tg^ mice, we blocked arginase activity in infected IL-13^tg^ mice. Because this arginase inhibition resulted in a decreased susceptibility to experimental Chagas disease our study supports in summary the conclusion that IL-13/IL-4Rα-driven Arg-1 expression contributes to the permissiveness of the host to *T. cruzi* infection.

## Introduction

*Trypanosoma cruzi*, a protozoan parasite that belongs to the family of Trypanosomatidae, is the causative agent of Chagas disease, a neglected tropical disease that is a major public health problem in Latin America (1). The complex lifecycle of *T. cruzi* involves several developmental stages in the insect vector and in vertebrates. In the vertebrate host, all nucleated cells can be infected by the parasite but macrophages are one of the most important cell types for *T. cruzi* replication during the acute phase of the infection. However, mechanisms that are involved in the permissiveness of host cells to *T. cruzi* infection are not completely understood so far.

A T helper type 1 (Th1) immune response characterized by the production of interferon-gamma (IFN-γ) is protective against experimental infection with *T. cruzi* (2–5) through the induction of classically activated macrophages (CAM). In these macrophages, the production of reactive nitrogen intermediates (RNI) by the activity of the inducible nitric oxide synthase 2 (NOS2) is central to the elimination of the intracellularly replicating amastigote developmental stage of *T. cruzi*. In contrast to a Th1 type immune response, the impact of a Th2-dominated immune response on the outcome of experimental Chagas disease is less clear. Th2 cells characterized by the secretion of interleukin (IL)-4 and IL-13 are only associated with an increased susceptibility to *T. cruzi* (6–10). However, downstream mechanisms that account for a Th2-mediated susceptibility after experimental infection with *T. cruzi* are not clearly established.

In general, the Th2-cytokines IL-4 and IL-13 mediate the development of alternatively activated macrophages (AAM) through binding to the IL-4 receptor-alpha (Rα) chain (11). Particularly, the induction of arginase (Arg)-1 in these cells is associated with an enhanced susceptibility to infection with parasites of the family Trypanosomatidae (12–15). In this context, there are two putative mechanisms by which IL-4Rα-induced Arg-1 may counteract effector functions against Trypanosomatidae in macrophages. At first, Arg-1 could antagonize the production of RNI by NOS2 either through depletion of L-arginine, the common substrate of these two enzymes, or by the inhibition of NOS2 through metabolites of the Arg-1 pathway (12–16). Furthermore, arginase activity results in the supply of polyamines capable to support the proliferation of intracellular parasites (15).

During experimental *Leishmania major* infection the IL-4Rα-mediated development of AAM and the associated strong Arg-1 induction account for the enhanced susceptibility of BALB/c mice (12, 13, 15, 17, 18). However, for infection with *T. cruzi* there is only proof based on *in vitro* experiments that the high arginase-activity in IL-4Rα-induced AAM supports the intracellular parasite replication by counteracting the antitrypanosomal effector mechanism mediated by NOS2 (19, 20). In experimental Chagas disease, however, it has been only shown that Arg-1 positive myeloid-derived suppressor cells (MDSC) infiltrate the heart of *T. cruzi* infected mice during the acute phase of the disease and that the degree of this infiltration correlates with the tissue parasite load (21, 22). Unfortunately, our knowledge about the function of AAM after infection with *T. cruzi in vivo* is rather limited but studies addressing the impact of IL-4Rα-induced Arg-1 on the permissiveness of host cells to this parasite are absolutely required because a human study points at a crucial role of the IL-4Rα for developing a cardiomyopathy, the major complication during the chronic phase of Chagas disease (23).

The poor expression of IL-4 and IL-13 after experimental infection of wildtype mice with *T. cruzi* nearly exclude a reasonable analysis of the contribution of the IL-4Rα-Arg-1 pathway in susceptibility to experimental Chagas disease. In the present study, we therefore took advantage of IL-13-overexpressing (^tg^) mice (24) and analyzed the outcome of *T. cruzi* infection under an increased Th2 immune response.

## Results

### Increased susceptibility of IL-13-overexpressing mice to *T. cruzi* infection

**Figure 1:**
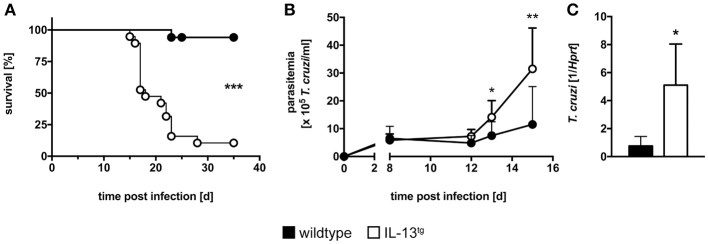
Increased susceptibility of infected IL-13^tg^ mice. Transgene-negative littermate control (closed symbols) and IL-13^tg^ (open symbols) mice were infected i.p. with 50 *T. cruzi* blood trypomastigotes for the analysis of parasitemia and survival and with 500 *T. cruzi* blood trypomastigotes for determination of the parasite tissue burden. **(A)** Survival and **(B)** parasitemia were assessed during infection. Results are expressed as the survival rate of 17–19 mice per group of two pooled experiments **(A)** or as mean parasitemia ±SD of 9–10 mice per group of one representative out of two experiments **(B)**. **(C)** Tissue parasite burden in the spleen was analyzed by quantitative real-time PCR at the time point “moribund” at which the first animal had to be euthanized (16 days post infection in this particular experiment). Results are expressed as the means ±SD of 4–6 mice per group of one representative out of two experiments. Statistical analysis was performed using the Log Rank test **(A)** or Mann Whitney *U* test **(B,C)** defining differences between IL-13^tg^ and wildtype mice as significant (^*^p ≤ 0.05; ^**^p ≤ 0.01, ^***^p ≤ 0.001).

To investigate the role of IL-13 during experimental *T. cruzi* infection IL-13^tg^ mice and wildtype littermates were infected i.p. with 50 blood trypomastigotes of the Tulahuen strain and the survival rate and parasite load during the acute phase of infection was determined (Figure 1). Compared to wildtype littermates, IL-13^tg^ mice were highly susceptible to a normally sublethal dose of *T. cruzi* with a median survival of 18 days p.i. (Figure 1A). Moreover, IL-13^tg^ mice developed a significantly higher parasitemia 13 and 15 days p.i. (Figure 1B). At the time point “moribund” at which the first infected animal had to be euthanized, the increased susceptibility of IL-13^tg^ mice to *T. cruzi* was also reflected by the significantly increased tissue parasitism in the spleen when compared to the tissue parasite load in wildtype littermates (Figure 1C). Hence, increased levels of IL-13 result in an accelerated susceptibility to *T. cruzi* infection.

### Histopathology in *T. cruzi*-infected IL-13^tg^ mice

**Figure 2:**
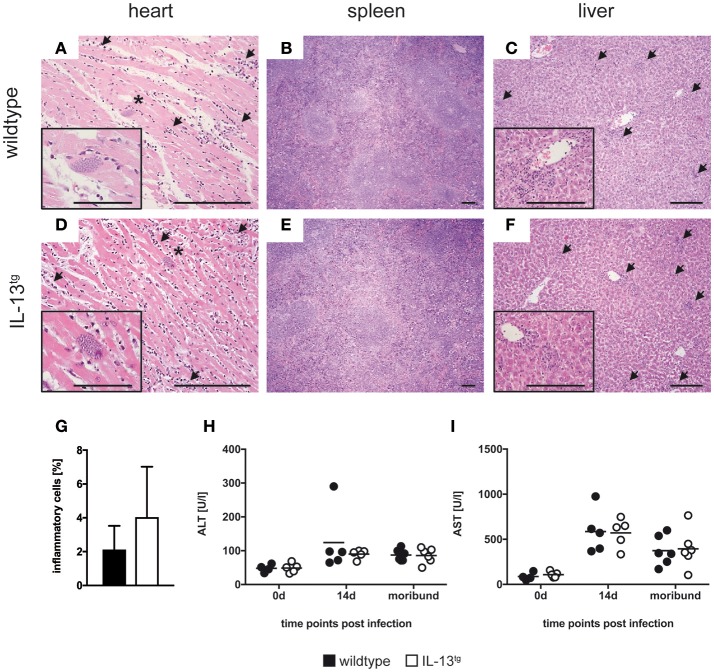
IL-13 has no influence on organ pathology during *T. cruzi* infection. Transgene-negative littermate control (closed symbols) and IL-13^tg^ (open symbols) mice were infected i.p. with 500 *T. cruzi* blood trypomastigotes. **(A–F)** Organ sections of heart **(A,D)**, spleen **(B,E)**, and liver **(C,F)** from wildtype **(A–C)** and IL-13^tg^ **(D–F)** mice were prepared at the time point “moribund” at which the first animal had to be euthanized (16 days post infection in this particular experiment) and stained with hematoxylin and eosin. Representative photomicrographs from 4 to 6 mice per group of one representative out of two experiments are shown (bar, 200 μm; arrow, infiltration; ^*^, parasite nest). **(G)** Quantification of heart inflammation as depicted in **(A)** and **(D)**. **(H,I)** Liver-derived enzymes ALT **(H)** and AST **(I)** were quantified in sera at the indicated time points after infection. Results are expressed as the means of 4–6 mice per group of one representative out of two experiments.

Mice often succumb to experimental Chagas disease after infection with reticulotropic strains due to a severe inflammation and subsequent immunopathology in infected organs (e.g., liver necrosis) (25, 26). To evaluate the degree of tissue damage in *T. cruzi*-infected wildtype and IL-13^tg^ mice, sections of paraffin-embedded tissue from heart (Figures 2A,D), spleen (Figures 2B,E), and liver (Figures 2C,F) were prepared and stained with hematoxylin-eosin. Overall, no significant differences in the degree of pathology in organs form wildtype and IL-13^tg^ mice were observed. However, quantification of heart inflammation revealed a tendentiously increased infiltration in cardiac tissue of mutant animals (Figure 2G). To quantify the extent of liver pathology after infection with *T. cruzi*, the content of the liver-derived enzymes ALT (Figure 2H) and AST (Figure 2I) were quantified in sera of infected mice. Both enzymes were found after infection in increasing quantities yet no differences between infected wildtype and IL-13^tg^ mice could be observed. Importantly, at the time point “moribund” at which the first infected animal had to be euthanized, the amounts of ALT and AST in the serum of dying IL-13^tg^ mice did not exceed those measured in surviving wildtype mice. Together, these data indicate that IL-13^tg^ mice do not succumb to an increased inflammation and immunopathology. The elevated parasite load rather appears to be responsible for a premature death in the presence of high levels of IL-13.

### Cytokine expression in *T. cruzi*-infected Il-13^tg^ mice

**Figure 3:**
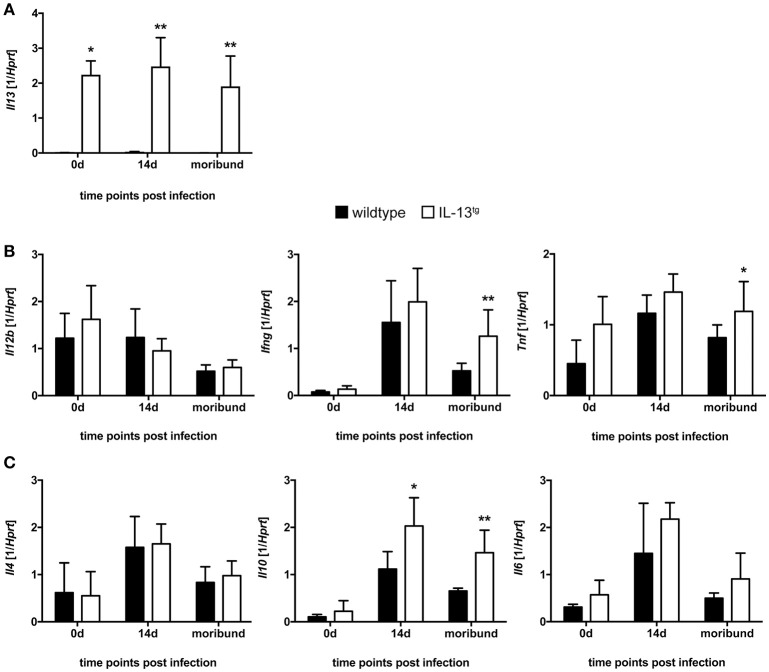
Cytokine expression in *T. cruzi*-infected wildtype and IL-13^tg^ mice. Transgene-negative littermate control (closed bars) and IL-13^tg^ (open bars) mice were infected i.p. with 500 *T. cruzi* blood trypomastigotes. mRNA expression of **(A)** *Il13*, **(B)** *Il12b, Ifng, Tnf*, **(C)** *Il4, Il10*, and *Il6* in spleens was quantified by real-time PCR at the indicated time points post infection (“moribund” indicates the time point at which the first animal had to be euthanized; 16 days post infection in this particular experiment). Results are expressed as the means ±SD of 4 - 6 mice per group of one representative out of two experiments. Statistical analysis was performed using the Mann Whitney *U* test defining differences between IL-13^tg^ and wildtype mice as significant (^*^p ≤ 0.05; ^**^p ≤ 0.01).

During experimental Chagas disease, cytokines are important for inducing a protective Th1 immune response and subsequent effector mechanisms in macrophages (2, 4). However, the release of these inflammatory mediators has to be tightly regulated to prevent an immunopathological sequelae (27). To examine the influence of IL-13 overexpression on the cytokine response during experimental Chagas disease, different pro-inflammatory and anti-inflammatory/regulatory cytokines were analyzed by quantitative real time RT-PCR in spleen homogenates of uninfected and *T. cruzi*-infected mice (Figure 3).

Although *Il13* gene expression was highly induced in spleens of IL-13^tg^ mice (Figure 3A), gene expression of the pro-inflammatory cytokines *Il12b, Ifng, Tnf* (Figure 3B) and the anti-inflammatory/regulatory cytokines and *Il4* and *Il6* (Figure 3C) were not significantly different between wildtype and IL-13^tg^ mice 14 days after *T. cruzi* infection. Only the anti-inflammatory cytokine *Il10* was significantly increased in spleen homogenates of IL-13^tg^ mice at this time point (Figure 3C). At the time point “moribund” at which the first infected animal had to be euthanized however, transcript levels of *Ifng* and *Tnf* were found to be significantly elevated in IL-13^tg^ (Figure 3B) and the significantly increased gene expression of *Il10* was still apparent in these mice (Figure 3C). Together, these results reveal that high amounts of IL-13 during *T. cruzi* infection are not accompanied by a biased cytokine response.

### NOS2 expression and RNI production in spleens of *T. cruzi*-infected IL-13^tg^ mice

**Figure 4:**
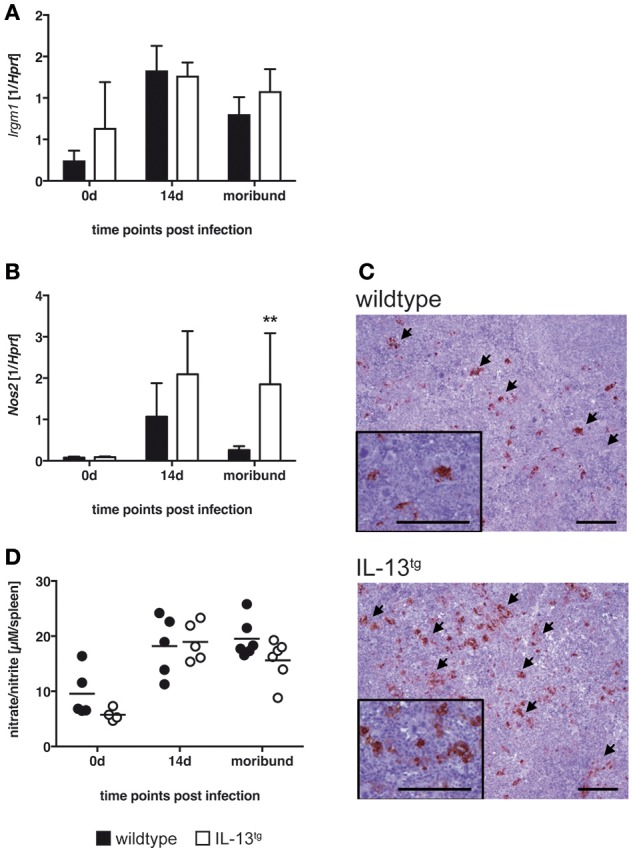
Classical macrophage activation is not altered in IL-13^tg^ mice after infection with *T. cruzi*. Transgene-negative littermate control (closed bars) and IL-13^tg^ (closed bars) mice were infected i.p. with 500 *T. cruzi* blood trypomastigotes. **(A,B)** mRNA expression of *Irgm* **(A)** and *Nos2* **(B)** in spleens were quantified by real-time PCR at the indicated time points post infection (“moribund” indicates the time point at which the first animal had to be euthanized; 16 days post infection in this particular experiment). Results are expressed as the means ±SD of 4–6 mice per group of one representative out of two experiments. Statistical analysis was performed using the Mann Whitney *U* test defining differences between IL-13^tg^ and wildtype mice as significant (^**^p ≤ 0.01). **(C)** For immunohistochemical analysis, spleens were isolated at the time point “moribund” at which the first animal had to be euthanized (16 days post infection in this particular experiment). Histological sections were stained for NOS2 and counterstained with hematoxylin. Representative photomicrographs from 4 to 6 mice per group of one representative out of two experiments are shown (bar, 200 μm; arrow, NOS2-positive cells). **(D)** Nitrate/nitrite levels in spleen homogenates were determined by the Griess reaction after reduction of nitrate to nitrite. Results are expressed as means of 4–6 mice per group of one experiment.

During experimental Chagas disease, LRG-47- and NOS2-dependent effector mechanisms in CAM are crucial to eliminate *T. cruzi* (2, 28). Because IL-13 is known to modulate the activation status of macrophages, LRG-47 and NOS2 expression as well as RNI production were analyzed in the spleen of *T. cruzi* infected wildtype and IL-13^tg^ mice (Figure 4).

Gene expression of *Irgm* was comparable in spleens of both, wildtype and IL-13^tg^ mice during the course of experimental Chagas disease (Figure 4A). *Nos2* gene expression was strongly induced in both mouse strains (Figure 4B). Thereafter, the quantity of *Nos2* transcripts declined in wildtype mice whereas in moribund IL-13^tg^ mice *Nos2* expression was significantly enhanced. Immunohistochemical staining of NOS2 revealed an enhanced appearance of this enzyme in spleens of moribund IL-13^tg^ mice when compared to corresponding wildtype mice (Figure 4C). Because NOS2 metabolizes L-arginine eventually to RNI which are effective against the intracellular parasite, we quantified the content of these reactive intermediates in homogenates of spleens isolated from uninfected and *T. cruzi*-infected wildtype and IL-13^tg^ mice (Figure 4D). After infection, the production of RNI was induced in spleens of both, wildtype and IL-13^tg^ mice. Thereafter, the amount of intermediates appeared to decline in spleen homogenates isolated from moribund IL-13^tg^ mice and were found to be slightly reduced compared to corresponding wildtype mice. Together, after infection with *T. cruzi* the expression of NOS2 was not impaired in the presence of high IL-13 concentrations. In contrast, NOS2 levels increased during the course of disease. However, this was not reflected by an also elevated production of RNI.

### IL-13 mediates alternatively activated macrophages in IL-13^tg^ mice

**Figure 5:**
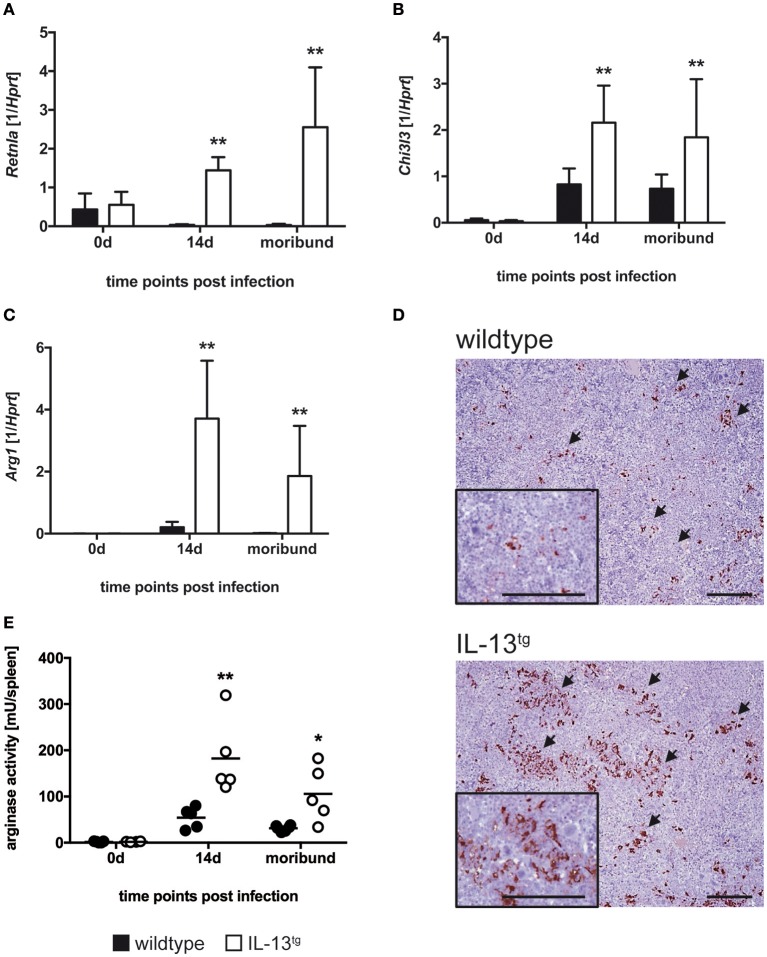
Increased alternative macrophage activation in *T. cruzi*-infected IL-13^tg^ mice. Transgene-negative littermate control (closed bars) and IL-13^tg^ (open bars) mice were infected i.p. with 500 *T. cruzi* blood trypomastigotes. **(A–C)** mRNA expression of *Retnla* **(A)**, *Chi3l3* **(B)**, and *Arg1* **(C)** in spleens were quantified by real-time PCR at the indicated time points post infection (“moribund” indicates the time point at which the first animal had to be euthanized; 16 days post infection in this particular experiment). Results are expressed as the means SD± of 4–6 mice per group of one representative out of two experiments. Statistical analysis was performed using the Mann Whitney *U* test defining differences between IL-13^tg^ and wildtype mice as significant (^**^p ≤ 0.010). **(D)** For immunohistochemical analysis, spleens were isolated at the time point “moribund” at which the first animal had to be euthanized (16 days post infection in this particular experiment). Histological sections were stained for Arg-1 and counterstained with hematoxylin. Representative photomicrographs from 4 to 6 mice per group of one representative out of two experiments are shown (bar, 200 μm; arrow, Arg-1-positive cells). **(E)** Arginase activity in spleen homogenates were determined by arginase assay at the indicated time points post infection (“moribund” indicates the time point at which the first animal had to be euthanized; 16 days post infection in this particular experiment). Results are expressed as means ±SD of 4–6 mice per group of one experiment. Statistical analysis was performed using the Mann Whitney U test defining differences between IL-13^tg^ and wildtype mice as significant (^*^p ≤ 0.05; ^**^p ≤ 0.01).

IL-13 induces an alternative activation of macrophages thereby favoring the growth of intracellular parasites (17, 29). AAM are characterized by enhanced expression of unique markers such as *Retnla, Chi3l3*, and *Arg1*. To evaluate whether these markers are induced in IL-13^tg^ mice after infection with *T. cruzi*, we performed quantitative real time RT-PCR of spleen homogenates (Figures 5A–C). Before infection, *Retnla* (Figure 5A) and *Chi3l3* (Figure 5B) gene expression was hardly detectable. After infection of wildtype mice with *T. cruzi, Retnla* was not expressed in wildtype mice whereas *Chi3l3* was induced to quantifiable levels. However, transcript levels of both *Retnla* (Figure 5A) and *Chi3l3* (Figure 5B) were significantly enhanced in spleens of infected IL-13^tg^ mice. *Arg1* was barely expressed in spleens of *T. cruzi*-infected wildtype mice (Figure 5C). Compared to this, *Arg1* gene expression was significantly increased approximately 12–18 fold in spleen homogenates prepared from infected IL-13^tg^ mice (Figure 5C). We next analyzed the expression of Arg-1 in spleens of moribund IL-13^tg^ mice and corresponding wildtype mice by immunohistochemistry (Figure 5D). Staining of Arg-1 disclosed a very strong appearance of the enzyme in IL-13^tg^ mice that was strikingly different from corresponding wildtype mice in which Arg-1 was hardly detectable (Figure 5D). To eventually examine arginase enzyme activity in wildtype and IL-13^tg^ mice during experimental Chagas disease, the urea concentration after L-arginine hydrolysis in spleen homogenates was quantified (Figure 5E). Before infection, no enzyme activity was detectable in spleens of both wildtype and IL-13^tg^ mice. After infection with *T. cruzi*, a rather low arginase activity was induced in wildtype mice but, in contrast, this activity was significantly increased approximately 4 fold in spleen homogenates of IL-13^tg^ mice.

### Arg-1 accounts for the increased susceptibility of il-13^tg^ mice to experimental chagas disease

**Figure 6:**
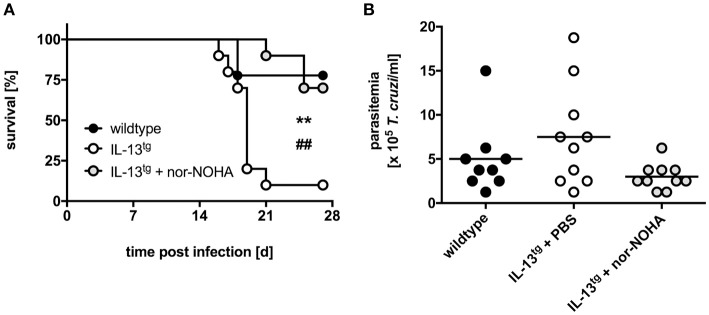
Inhibition of Arg-1 results in a decreased susceptibility of infected IL-13^tg^ mice to *T. cruzi* infection. Transgene-negative littermate control and IL-13^tg^ mice were infected i.p. with 50 *T. cruzi* blood trypomastigotes for the analysis of parasitemia and survival. Arginase activity was inhibited in IL-13^tg^ mice by daily i.p. administration with 600 μg nor-NOHA. PBS-treated IL-13^tg^ mice and wildtype mice served as control groups. **(A)** Survival during the course of infection and **(B)** parasitemia at 16 days of infection. Results are expressed as percent survival **(A)** and mean parasitemia **(B)** of 9–10 mice per group of two pooled experiments. Statistical analysis was performed using the Log Rank test defining differences between IL-13^tg^ and wildtype mice (^*^,^**^ *p* ≤ 0.01) and between IL-13^tg^ and IL-13^tg^ mice treated with nor-NOHA (#,##*p* ≤ 0.01) as significant.

So far, we have shown that during experimental Chagas disease overexpression of IL-13 resulted in high Arg-1 expression and arginase activity that was associated with a decreased survival rate and enhanced parasitemia. Because Arg-1 is implicated in the impairment of effector functions in *T. cruzi*-infected macrophages *in vitro* (20), we next sought to demonstrate that Arg-1 in fact accounted for the increased susceptibility of IL-13^tg^ mice to *T. cruzi* infection. We therefore infected wildtype and IL-13^tg^ mice with *T. cruzi*, treated transgenic mice either with PBS or with the arginase inhibitor N^ω^-hydroxy-nor-arginine (nor-NOHA) and monitored survival and determined parasitemia (Figure 6). *T. cruzi*-infected IL-13^tg^ mice reached a median survival time of 19 days (Figure 6A). In contrast, the median survival time of infected nor-NOHA-treated IL-13^tg^ mice was extended to 28 days. In addition to the survival rate, the parasitemia was determined at the time point “moribund” at which the first infected animal had to be euthanized (Figure 6B). The concentration of *T. cruzi* in the blood of infected wildtype mice was approximately 5 × 10^5^ trypomastigotes/ml and was with 7.5 × 10^5^ trypomastigotes/ml increased in IL-13^tg^ mice. Although not statistically significant different, Arg-1 inhibition resulted in a decrease in parasitemia of IL-13^tg^ to 3 × 10^5^ trypomastigotes/ml (Figure 6B). Hence, Arg-1 appears to be a key factor induced by IL-13 that mediates susceptibility to *T. cruzi* in IL-13^tg^ mice.

## Discussion

A Th2 immune response accompanied by AAM and Arg-1 is assumed to undermine the protective Th1 immune response against infections with trypanosmatids and thus contributes to susceptibility. In contrast to the well characterized model system for cutaneous leishmaniasis not much is known about the impact of the IL-4Rα-Arg-1 axis on the course of experimental Chagas disease. However, a human study points toward a crucial role of the IL-4Rα for developing of cardiomyopathy during Chagas disease, the major complication during the chronic phase of the infection (23). So far, most studies addressing the function of IL-4Rα-mediated signaling in experimental Chagas disease employed IL-4-deficient^(−/−)^ and STAT6^−/−^ mice or neutralization of IL-4 by administration of monoclonal antibodies (7, 10, 30, 31). However, these reports revealed conflicting results with respect to IL-4Rα as a susceptibility factor during infection with *T. cruzi*. Because the induction of the IL-4Rα ligands IL-4 and IL-13 is rather low the analysis of the outcome of *T. cruzi* infection in IL-4^−/−^ and STAT6^−/−^ or under anti-IL-4 treatment is limited. Hence, to determine the hypothetical effect of the IL-4Ra on the outcome of Chagas disease (23) we infected mice with *T. cruzi* which overexpressed IL-13 specifically in T cells (24). Our study is the first that clearly shows a contribution of the IL-4Rα ligand IL-13 to the susceptibility to experimental Chagas disease *in vivo*. As it has been shown for cutaneous leishmaniasis (29), *T. cruzi*-infected IL-13^tg^ mice presented an increased parasite replication accompanied by an enhanced mortality compared to infected wildtype mice. Moreover, the support of the parasite growth by IL-13 appeared to be a crucial mechanism for the increased susceptibility.

The effective immune response against *T. cruzi* is initiated by a pro-inflammatory Th1 immune response leading to high amounts of IFNγ which in turn induces effector responses in CAM such as LRG-47 and NOS2 (2, 28). Hence, the common underlying mechanism of an increased susceptibility to *T. cruzi* infection is thought to be an impaired release of pro-inflammatory mediators such as IL-12 (4) and a reduced production of IFNγ (2). Therefore, in our study IL-13 might have suppressed this pro-inflammatory immune response leading to an insufficient IFNγ production and subsequent impaired CAM induction. However, we did not observe this kind of IL-13-mediated suppression of protective Th1 cytokines. To the contrary, the release of pro-inflammatory cytokines in the spleen of *T. cruzi*-infected IL-13^tg^ mice was rather increased. Accordingly, the IFNγ/TNF-mediated expression of LRG-47 was similar and the expression of NOS2 was even enhanced in IL-13-overexpressing mice. However, this increased enzyme expression did not result in an also elevated release of RNI by CAM in IL-13^tg^ mice during experimental Chagas disease.

An exacerbated inflammatory immune response also contributes to immunopathology during experimental Chagas disease which is controlled by IL-10 (27, 32). Because in the present study overexpression of IL-13 also resulted in a significantly augmented expression of IL-10, an inflammation-induced pathology mediated by the increased release of the Th1 cytokines IFNγ and TNF appears to be greatly counterregulated even though the cellular infiltration in cardiac tissue was tendentiously increased. Hence, the amplified levels of IL-10 may account for the largely checked inflammatory tissue inflammation in *T. cruzi*-infected IL-13^tg^ mice. Taken together, IL-13 overexpression did not negatively affect the generation of NOS2-dependent effector responses in CAM, the pro-inflammatory cytokine release and inflammation during experimental Chagas disease.

Whereas classical macrophage activation was rather similar in *T. cruzi*-infected IL-13^tg^ and wildtype mice, overexpression of IL-13 lead to an elevated induction of markers for alternative macrophage activation with a strikingly increased Arg-1 production and enhanced arginase activity. Hence, our study confirms, that during infection IL-13 promotes a strong alternative macrophage activation (29, 33, 34). Additionally, the increased levels of IL-10 in *T. cruzi*-infected IL-13^tg^ mice may add to alternative macrophage activation in these animals as this cytokine is also able to induce Arg-1 directly or through the upregulation of the IL-4Rα on macrophages (35, 36). Because the induction of AAM could create optimal conditions for an uncontrolled parasite proliferation by the effects of Arg-1 (15, 18), we examined the overall impact of arginase activity on the susceptibility of IL-13^tg^ mice to *T. cruzi* infection and inhibited the enzyme by administering nor-NOHA. This treatment reduced arginase activity in infected IL-13^tg^ mice to levels observed in corresponding wildtype animals (data not shown) and significantly ameliorated the outcome of experimental Chagas disease when compared to untreated IL-13^tg^ mice. Hence, Arg-1 appears to be a key molecule that mediates the IL-13-dependent susceptibility to *T. cruzi* infection.

There are several mechanisms by which Arg-1 may undermine protective immune responses against intracellular parasites. IL-13 induces via the IL-13/IL-4 type II receptor and STAT6 Arg-1 in AAM but also in MDSC which has also been described to promote susceptibility to *T. cruzi* infection (21, 37). The fact that Arg-1 and NOS2 coexist in the here examined *T. cruzi*-infected IL-13^tg^ mice may favor a role of MDSC in the outcome of infection. The suppressive mechanisms of MDSC e.g. by local L-arginine consumption appear to affect T cells (38, 39). Because both enzymes Arg-1 and NOS2 metabolizes L-arginine, the suppressive capacity of MDSCs depends on IL-4Rα-mediated Arg-1 and/or IFNγ-induced NOS2 activities (38, 40, 41). Although we have not directly addressed the cellular source of IFNγ, the phenotype of MDSC or the T cell response in *T. cruzi*-infected IL-13^tg^ mice, the simultaneous occurrence of increased NOS2 and Arg-1 indicates that MDSCs contribute to the susceptibility of IL-13^tg^ mice to experimental Chagas disease.

In addition to a suppressive effect of L-arginin depletion on T cells by MDSCs, IL-13-induced Arg-1 may also have a direct effect on parasite replication in host cells like macrophages. Accordingly, macrophages with elevated arginase activity, induced through the IL-4Rα or the *T. cruzi* component cruzipain, promote parasite growth which could be inhibited by blockade of enzyme activity (19, 20).

In host cells, Arg-1 induction can support parasite replication by competitive depletion of the common substrate L-arginine which is also used by NOS2 to generate RNI (42). Hence, although NOS2 is elevated in *T. cruzi*-infected IL-13^tg^ mice the simultaneously enhanced IL-4Rα-mediated arginase activity may explain the rather reduced levels of RNI by an overall increased consumption of L-arginine. Along this line, IL-4Rα ligation has recently been shown to induce asymmetric dimethylarginine (ADMA) (43), which represents an endogenous inhibitor of NOS2 activity (44). Because experimental *T. cruzi* infection of mice results in increased levels of ADMA concomitant with a reduced NOS2 activity (45) the IL-13-mediated induction of this methylated derivate of L-arginine may - although independent of Arg-1 - also inhibit effector responses in macrophages.

Additionally, arginase activity results in the production of polyamines, which appear to be essential for intracellular parasite replication (15). Parasites are able to generate polyamines endogenously but can also utilize to a great extent host polyamines. They are synthesized by metabolic processes that are similar in parasites and the host including arginase, which catalyzes the enzymatic hydrolysis of L-arginine to L-ornithine and urea, and ornithine decarboxylase, which catalyzes the enzymatic decarboxylation of L-ornithine in putrescine (46, 47). In turn, putrescine is a substrate for spermidine synthase to synthetize spermidine which is essential for growth and survival of trypanosomatid parasites. Hence, within AAM the microenvironment with an increased activity of host Arg-1 and subsequent elevated levels of host polyamines represent a “land of plenty” for intracellular parasites. Additionally, trypanosomatid parasites have a unique metabolism, in which spermidine is further metabolized by trypanothione reductase which provides an intracellular antioxidant system essential to survive the hostile intracellular environment (46–48). Together, during infection with *T. cruzi* IL-13 induces an increased arginase activity and subsequently elevated amounts of polyamines. This excess supply of host polyamines may also account for the arginase-dependent uncontrolled parasite replication in *T. cruzi*-infected IL-13^tg^ mice.

In summary, we here give evidence that IL-4Rα-induced Arg-1 mediates susceptibility to acute experimental Chagas disease by several - mutually not exclusive - mechanisms downstream of Arg-1 including L-arginine depletion in MDSCs and AAM and polyamine synthesis. A correlation between Arg-1, arginase activity and susceptibility was already shown in patients with visceral leishmaniasis (49, 50). However, although there is a clear link of IL4RA polymorphism and cardiomyopathy in human Chagas disease (23), the impact of Arg-1 and the subsequent functional effects have not been investigated so far. Nevertheless, such studies will possibly unravel novel therapeutic targets.

## Methods

### Mice and parasites

IL-13^tg^ mice on a BABL/c background were kindly provided by Andrew McKenzie (MRC Laboratory of Molecular Biology, Cambridge, UK) and bred under specific-pathogen-free conditions in the animal facility of the Research Center Borstel. For experiments, female transgenic mice and transgene-negative littermates aged 6 to 8 weeks were infected intraperitoneally (i.p.) with the *T. cruzi* strain Tulahuen (WHO reference strain M/HOM/CH/00/Tulahuen C2). To obtain high numbers of *T. cruzi* blood form trypomastigotes for infection experiments and to prevent the transfer of lymphocytes and antibodies, SCID mice mice (purchased from Charles River, Sulzfeld, Germany) were i.p. infected with 1 × 10^7^ parasites in cryopreserved *T. cruzi* stabilates. At day 12 post infection, blood was collected from infected SCID, mixed with heparin and parasites were enriched in the plasma by differential centrifugation. Parasites were resuspended in PBS/0.5% glucose and used for infection. An infection dose of 500 blood trypomastigotes was used to induce detectable inflammatory cytokine responses and pathology. For determining parasitemia and mortality, a sublethal dose of 50 parasites was used (26, 51). For arginase-1 inhibition, mice were treated daily with 600 μg/mouse Nor-NOHA (N(omega)-hydroxy-l-arginine; Alexis Biochemicals) in 200 μl PBS starting 2 days before infection. Control animals were treated with the same amount of PBS. During infection experiments, mice were kept under barrier conditions in the BSL 3 facility at the Research Center Borstel in individually ventilated cages. All experiments were conducted according to the German animal protection laws and were approved by the Animal Research Ethics Board of the Ministry of Environment, Kiel, Germany.

### Determination of parasitemia and tissue parasite load

Blood parasitemia was determined in 3 μl of tail vein blood that was lyzed in 27 μl NH_4_Cl (0.87% [wt/vol]). Viable parasites were counted in a Neubauer chamber.

Tissue parasite burdens were analyzed by quantitative real-time PCR of genomic DNA isolated from the spleen. A 70 bp sequence of the 140/116-kDa antigen gene of T. cruzi (accession no. U15616) was amplified with the forward primer 5-ACT CAT CGG GTT TGA AGC AT-3, the reverse primer 5-GCC AGG GTC TAG TAC TCT TTG CT-3, and the internal probe 5-CAG CAG GC-3_ A 107 bp stretch of the murine hypoxanthine–guanine phosphoribosyltransferase (*hprt*) gene, used for quantification of host DNA, was amplified with the forward primer 5-GTG GCC CTC TGT GTG CTC-3, the reverse primer 5-TCT ACA GTC ATA GGA ATG GAT CTA TCA-3, and the internal probe 5-ACC TGC TG-3. Quantitative PCR was performed on a Light Cycler 480 (Roche Diagnostics Corporation, Indianapolis, IN). Data were analyzed employing the “Second Derivative Maximum” and “Standard Curve Method.”

### Histopathology and immunohistochemistry

For histolopathological and immunohistochemical analysis, heart, spleen and liver were isolated at the time point “moribund” at which the first animal had to be euthanized and fixed in 4% formalin/PBS, set in paraffin blocks and sectioned (2 μm). Histopathological analyses were performed using standard protocols for hematoxylin/eosin staining. For quantification of heart inflammation, serial sections of heart tissue were analyzed for the degree of cellular infiltaraion using the ImageJ software (NIH, Bethesda, Maryland) as described (52). For NOS2 staining, tissue sections were stained with a polyclonal rabbit anti-mouse NOS2 antiserum (Upstate) as previously described (53). For Arg-1 staining, sections were stained using a mouse anti-mouse arginase-1 antibody (BD Transduction) and the Vector M.O.M. immunodetection kit (Vector Laboratories).

### AST and ALT assay

Serum was prepared using the BD Microtainer SST Tubes (BD Pharmingen) and the aspartate transaminase (AST), and alanine transaminase (ALT) activity was determined by using the Reflotron System of Diagnosis (Roche Diagnostics).

### Quantitative real-time PCR

Organs were homogenized in 4M guanidinium-isothiocyanate buffer and total RNA was extracted by acid phenol extraction. cDNA was obtained using murine moloney leukemia virus reverse transcriptase (RvertAid, Invitrogen) and oligo-dT (12-18mer; Sigma) as a primer. Quantitative PCR was performed on a Light Cycler 480 Instrument (Roche Diagnostics). Data were analyzed employing the “Second Derivative Maximum” and “Standard Curve Method” using hypoxanthine-guanine phosphoribosyltransferase (*hprt*) as a housekeeping gene to calculate the level of gene expression in relation to *hprt*. The following primer and probe sets were employed: *Arg1*: sense 5′-CCT GGA ACT GAA AGG AAA G-3′, antisense 5′-TTG GCA GAT ATG CAG GGA GT-3′, probe 2; *Chi3l3:* 5′-GAA CAC TGA GCT AAA AAC TCT CCT G-3′, antisense 5′- GAG ACC ATG GCA CTG AAC G-3′, probe 88; *Hprt*: sense 5′-TCC TCC TCA GAC CGC TTT T-3′, antisense 5′-CCT GGT TCA TCA TCG CTA ATC-3′, probe 95; *Ifng*: sense 5′-ATC TGG AGG AAC TGG CAA AA-3′, antisense 5′-TTC AAG ACT TCA AAG AGT CTG AGG TA-3′, probe 21; *Il4: sense* 5′-CAT CGG CAT TTT GAA CGA G-3′, antisense 5′-CGA GCT CAC TCT CTG TGG TG-3′, probe 2; *Il6: sense* 5′- CT ACC AAA CTG GAT ATA ATC AGG A-3′, antisense 5′-CCA GGT AGC TAT GGT ACT CCA-′, probe 6; *Il12b*: sense 5′-CCA TCA GCA GAT CAT TCT AGA CAA-3′, antisense 5′-CGC CAT TAT GAT TCA GAG ACT G-3′, probe 78; *Il13*: sense 5′-CCT CTG ACC CTT AAG GAG CTT AT-3′, antisense 5′-CGT TGC ACA GGG GAG TCT-3′, probe 17; *Irgm*: sens 5′-AAG GCC ACT AAC ATC GAA TCA-3′, antisense 5′-TGC CTT ATC TCA CTT AAT ACT CCT-3′, probe 82; *Nos2*: sense 5′-CTT TGC CAC GGA CGA GAC-3′, antisense 5′-TCA TTG TAC TCT GAG GGC TGA C-3′, probe 13; *Retnla*: sense 5′-TAT GAA CAG ATG GGC CTC CT-3′, antisense 5′-GGC AGT TGC AAG TAT CTC CT-3′, probe 3; *Tnf* : sense 5′-TGC CTA TGT CTC AGC CTC TTC-3′, antisense 5′-GAG GCC ATT TGG GAA CTT CT-3′, probe 49 (all from Roche).

### Arginase activity and nitrate production in spleen homogenates

To detect reactive nitrogen intermediates (RNI) in uninfected and infected mice, spleen homogenates were collected at different time points. After deproteination of homogenates using Micron YM-30 centrifugal filters (Millipore, Schwalbach, Germany), NO_3_ was converted into NO2 utilizing a commercial nitrate reductase kit (Cayman; Axxora, Lörrach, Germany). After adding Griess reagents, the content of NO_2_ was determined by photometric measurement reading the absorbance at 540 nm on a microplate reader (Sunrise; Tecan, Männedorf, Switzerland) as previously described (54). To determine arginase activity in murine tissue, weighed pieces of organs were homogenized in 100 μl of 0.1% Triton X-100 (Sigma) containing a protease inhibitor cocktail (Roche). Fifty microliter of 10 mM MnCl2 (Merck, Darmstadt, Germany) and 50 mM Tris-HCl (Merck) were added to all samples and the enzyme was activated by heating for 10 min at 55°C. Arginine hydrolysis was conducted by incubating 25 μl of the activated lysate with 25 μl of 0.5 M L-arginine (Merck) at 37°C for 60 min. The reaction was stopped with 400 μl of H_2_SO_4_ (96%)/H3PO4 (85%)/H2O (1/3/7, v/v/v). As a degree of arginase activity, the urea concentration was measured at 540 nm after addition of 25 μl α-isonitrosopropiophenone (Sigma; dissolved in 100% ethanol) followed by heating at 95°C for 45 min. One unit of arginase activity is defined as the amount of enzyme that catalyzes the formation of 1 μmol urea/min.

### Statistical analysis

Quantifiable data are expressed as means of individual determinations and standard deviation. Statistical analysis was performed using the unpaired Student's *t*-test, the Mann-Whitney *U* test, the Kruskal-Wallis test or the Log-Rank test defining different error probabilities (^*^p ≤ 0.05; ^**^p ≤ 0.01; ^***^p ≤ 0.001).

## Author contributions

MA performed the experiments, analyzed the data, and wrote the manuscript; CH conceived and designed the experiments and wrote the manuscript.

### Conflict of interest statement

The authors declare that the research was conducted in the absence of any commercial or financial relationships that could be construed as a potential conflict of interest.
